# The Effect of Arthrocentesis Treatment for Maximum Mouth Opening and Pain in Temporomandibular Joint Diseases and the Effect of Splint, Drug, and Physical Therapy on This Treatment

**DOI:** 10.3390/medicina59101767

**Published:** 2023-10-04

**Authors:** Mehmet Gökhan Demir

**Affiliations:** Department of Oral and Maxillofacial Surgery, Istanbul Medical School, Istanbul University, 34452 İstanbul, Türkiye; mgokhandemir@yahoo.com

**Keywords:** arthrocentesis, temporomandibular joint disorder, splint therapy, temporomandibular disc displacement, physical therapy

## Abstract

*Background and Purpose*: Temporomandibular disorders (TMD) are a heterogeneous group of musculoskeletal and neuromuscular diseases involving the temporomandibular joint complex and the surrounding muscle and osseous structure. TMD can be classified as intra-articular or extra-articular. The aim of this study was to evaluate the effect of arthrocentesis in terms of maximum mouth opening (MMO) and pain in patients with TMD of intra-articular origin. In addition to this treatment, the effects of factors such as splints, medication, and physical therapy on arthrocentesis were examined. *Material and methods*: This retrospectively designed study was conducted with 79 patients who had previously undergone arthrocentesis. These patients were divided into three groups according to the Research Diagnostic Criteria for temporomandibular disorder: disc displacement (DD) with locking (Group 1), DD without locking (Group 2), and degenerative joint diseases (Group 3) groups. The maximum mouth opening (MMO) and visual analog score (VAS) values of the groups were recorded before arthrocentesis (Baseline: T0), on the third day after arthrocentesis (T1), and at the sixth month (T2) after arthrocentesis. Information about whether the patients received concurrent medical treatment, splint treatment, and physical therapy was also recorded. These data were compared between groups. *Results*: It was observed that the VAS scores in all three groups decreased from T1 compared to T0 (*p* < 0.05). Likewise, the MMO value increased in all groups at T1 compared to T0. (*p* < 0.05). It was observed that splint treatment, pain killer and muscle relaxant treatment, and physical therapy made no additional contribution to arthrocentesis in terms of reducing pain or increasing MMO value (*p* > 0.05). *Conclusions*: Arthrocentesis was observed to be effective in terms of pain and function in TMJ patients in this study. It was observed that splint therapy, physical therapy, and medical therapy made no additional contribution to arthrocentesis in terms of MMO or pain.

## 1. Introduction

Temporomandibular joint diseases (TMDs) are musculoskeletal dysfunctions and affect the temporomandibular joint (TMJ) and masticatory muscles. In this group of diseases, temporomandibular joint diseases include diseases that cause mechanical problems [1,2,3]. TMJ diseases are observed more frequently in women between the ages of 20 and 40. It is also known that they can be observed in all age groups and genders. Many complaints, such as jaw pain, clicking sounds, limitation of mouth opening, deviation, locking, otalgia, and headache, may be reported by patients with TMD diseases [4,5,6].

Temporomandibular diseases can be divided into two groups, depending on whether they are of intra-articular or extra-articular origin. Articular origin TMD can be classified into six groups, according to the Research Diagnostic Criteria for Temporomandibular Disorders (RDC/TMD). These are “disc displacement with reduction”, “disc displacement with reduction with intermittent locking”, “disc displacement without reduction with limited opening”, “disc displacement without reduction, without limited opening”, “degenerative joint disease”, and “subluxation” [7].

In the treatment of TMJ diseases, medical conservative treatment methods are primarily chosen. These may include soft diet, muscle relaxants, pain killer and anti-inflammatory drugs, occlusal splints, and physical therapy. When conservative treatment does not help, surgical methods are preferred [8]. Of these, the first method applied is arthrocentesis. In addition, methods such as arthroscopy, open surgery, and total joint replacement can be applied [8,9].

TMJ arthrocentesis was first described by Nitzan et al. [10]. Arthrocentesis helps to reduce inflammatory mediators, open intra-articular adhesions, and relax the articular disc. It has been shown in previous studies that arthrocentesis treatment is effective at reducing pain and increasing mouth opening in individuals with disc displacement [10,11,12].

Splint therapy is used in individuals with temporomandibular joint problems. Splints reduce the load by stabilizing the joint, thus reducing joint-related pain. The number of currently available studies is insufficient in terms of indicating the extent to which splint therapy and arthrocentesis are beneficial in TMJ diseases. On the other hand, a small number of studies have reported that they are beneficial [11,12].

The presence of muscle spasms and inflammatory mediators in the joint causes pain and limitations of the patient’s mouth opening. For this purpose, muscle relaxants and anti-inflammatory drugs are used in TMJ diseases. Their effect is limited to the acute stage of TMJ diseases.

Physical therapy is also beneficial for those with TMJ disease. Physical therapy has effects related to both pain control and increasing the range of motion of the joint. In this respect, giving patients physical therapy with a splint can contribute to their treatment [13].

The aim of this study is to examine arthrocentesis treatment in terms of maximum mouth opening (MMO) and pain in patients with temporomandibular joint disease. To achieve this, third-day short-term and sixth-month long-term data will be compared with the baseline data. Whether the additional splint, medical treatment, and physical therapy received by the concurrent patients made an additional contribution to arthrocentesis will be evaluated.

## 2. Materials and Methods

This retrospectively designed study was carried out on 79 patients, aged 18–50 years, who were admitted to the oral maxillofacial surgery outpatient clinic of the University Hospital between 2019 and 2022 and diagnosed with disc displacement with reduction, disc displacement without reduction, and degenerative joint disease. All of the data were collected from patient medical records. This study was approved by the ethical committee, and all of the patients gave their informed consent before this study. TMJ arthrocentesis was performed by a senior maxillofacial surgeon under local anesthesia. Maximum mouth opening (MMO) and pain scale were measured and recorded before arthrocentesis (baseline: T0), 3 days after arthrocentesis (T1), and 6 months after arthrocentesis (T2). Whether splint therapy, physical therapy, muscle relaxants, and anti-inflammatory drug treatment made a difference after arthrocentesis was also examined.

Patients with temporomandibular joint disease complaints and physical examination findings who did not respond to medical treatment were included in this study. The research diagnostic criteria for temporomandibular disorders (RDC/TMD) were used to diagnose the patients.

Patients were divided into the following three groups:

Group 1. Disc Displacement (DD) with locking group—DD with locking

(RDC/TMD criteria; Disc Displacement without reduction with locking-closed lock).

Group 2. Disc Displacement (DD) without locking group—DD without locking

(RDC/TMD criteria, including diagnosis, disc displacement with reduction, disc displacement with reduction with intermittent locking, and disc displacement without reduction without locking).

Group 3. Degenerative joint disease group—DJD.

Patients under the age of 18 and patients with previous surgical procedures to the temporomandibular region, previous arthrocentesis, orthognathic surgery, maxillofacial fracture, malignancy, radiotherapy to the head and neck region, history of inflammatory disease, and TMJ ankylosis were excluded from this study.

Arthrocentesis procedure:

Before the operation, the patient’s maximum mouth opening (MMO) and visual analog scale (VAS) score in terms of pain were recorded. The MMO value was measured by taking the average of three consecutive measurements of the distance between the maxilla and mandibula mid-central incisive teeth with the Digital Vernier caliper after the patients were asked to open their mouths as much as they could.

The VAS score, on the other hand, takes a value between 0 and 10 points. A score of 0 was defined as no pain, while 10 was defined as maximum pain. VAS scores were collected at T0, T1, and T2 for each patient.

After the surgical field disinfection procedure, the upper joint space method was applied [14,15]. After local anesthesia was applied to the auriculotemporal region, the line drawn between the middle height of the tragus and the outer corner of the eye with a 26 G needle, the anterior joint space was reached by entering approximately 1 cm anterior to the tragus. Control was provided with 2 mL of 0.9% NaCl solution to determine that the joint space was reached. Control was provided by the return of the liquid after it was given. Afterward, the second needle was inserted by entering the articular eminence. Arthrocentesis was completed with 500 cc of 0.9% NaCl solution. After the procedure, 1 mL of hyaluronic acid solution is given to the intra-articular space.

After the operation, the patient was discharged with the recommendation of a soft diet. The patient was called for control on the third day (T1) and for control on the 6th month (T2). The MMO and VAS scores were re-recorded on both control dates.

### 2.1. Splint Treatment

Splint therapy was recommended for all patients. However, some patients did not want to use splint therapy; therefore, those who received splint therapy were identified from the records.

A rigid splint for the lower jaw was proposed as a repositioning occlusal splint. The patients were asked to use the splint for 8 h each night for 6 months at night.

### 2.2. Muscle Relaxant and Anti-Inflammatory

In the case of joint pain and muscle tension after arthrocentesis, 6 mg tizanidine hydrochloride once a day and 600 mg etodolac once a day were prescribed. The treatment was given postoperatively for 7 days and was not extended thereafter.

### 2.3. Physical Therapy

Physical therapy was recommended for all patients. However, the TMJ patients who received physical therapy, according to their records, were noted in the study.

### 2.4. Statistical Evaluation

SPSS 23.0 (SPSS Inc. Chicago, IL, USA) was used in the evaluation. The paired *t*-test was used as a result of arthrocentesis, and the variables in arthrocentesis were analyzed with a *t*-test and ANOVA. *p* < 0.05 was considered statistically significant.

## 3. Results

This study included 79 patients who underwent arthrocentesis. Arthrocentesis was applied to 65 patients on the right side and 14 on the left side. All patients had unilateral TMJ disease. None of the patients who underwent arthrocentesis developed any complications such as facial paralysis, bleeding, hematoma, or local infection. Also, there were no reported complications due to physical therapy, splint therapy, and medical therapy.

The mean age of the patients was 35.4 ± 4 years for the men and 36.7 ± 6.1 years for the women.

Both groups were similar in terms of the MMO results in T0, T1, and T2 (*p* > 0.05) (Table 1).

While the MMO value was 36.1 at baseline (T0), it became 41.4 at T1 and 41.3 at T2. This value showed a significant increase compared to T0, both at T1 and T2 (*p* < 0.05). In terms of the MMO value, T1 and T2 did not differ statistically (*p* > 0.05) (Table 2).

While the VAS value was 6.2 at T0, this value decreased to 2.3 at T1 and 2.1 at T2 (*p* < 0.05). There was no significant difference between T1 and T2 (*p* > 0.05) (Table 2).

While the VAS score of those using arthrocentesis and splint was 5.11 at T0, this value was 1.76 at T1 and 1.87 at T2. In patients who did not receive splint treatment, the VAS score was 4.41 at T0, while this value was 1.87 at T1 and 1.90 at T2. There was no additional contribution of splint treatment in terms of the VAS score (*p* > 0.05) (Table 3 and Table 4).

The patients who used arthrocentesis and splint treatment had an MMO value of 36.9 at T0. That value was 42.1 at T1 and 42.6 at T2.

In those who did not use splints, the MMO value was 36.1 at the T0, 41.8 at the T1, and 43.2 at the T2.

It was observed that the splint treatment did not have an additional contribution or effect on the MMO value. (*p* > 0.05) (Table 5 and Table 6).

In Group 1, Group 2, and Group 3 patients, the MMO value increased significantly at T1 and T2 (*p* < 0.05) (Table 5 and Table 6). This difference manifested itself as a decrease in the VAS scores (*p* < 0.05) (Table 3 and Table 4). There was no difference between the scores of Group 1, Group 2, and Group 3 according to T0, T1, and T2 (*p* > 0.05).

In this study’s groups, no significant contribution or effect was observed in the MMO and VAS scores in terms of the use of muscle relaxants and anti-inflammatory drugs. (Table 3, Table 4, Table 5 and Table 6).

In terms of physical therapy, an increase in MMO values and a decrease in VAS scores were observed in those who received and did not receive physical therapy, respectively (*p* < 0.05). However, no significant change was found in the controls of both groups at T0, T1, and T2 (*p* > 0.05) (Table 3, Table 4, Table 5 and Table 6).

Whether the locking period was longer or shorter than 6 months did not make a difference in terms of the MMO value and VAS score (*p* > 0.05). However, it was observed that the MMO value increased, and the VAS score decreased both at T1 and T2 (*p* < 0.05) (Table 3, Table 4, Table 5 and Table 6).

## 4. Discussion

Internal derangement in the temporomandibular joint includes processes such as inflammation in the articular structures, changes in the intra-articular synovial fluid composition, pressure changes in the joint, adhesions, and disc derangement [12,16]. In cases where this process exceeds the self-healing capacity of the joint, structural changes occur and cause clinical findings.

For this purpose, patients were given splint treatments. However, their effects on the inflammatory process in the joint and in disc displacement without reduction have been the subject of discussion [17]. It has been shown that patients benefit from arthrocentesis treatment in such cases [18].

Arthrocentesis is a minimally invasive surgical procedure and contributes to the removal of intra-articular inflammatory mediators, the opening of adhesions, and the removal of free intra-articular particles [19,20,21]. Arthrocentesis was applied to 79 patients in this study. A decrease in VAS scores characterizing pain was observed in all of the DD with locking (Group 1), DD without locking (Group 2), and DJD (Group 3) groups. In addition, an increase was observed in the MMO values measured 3 days after arthrocentesis (T1). It was determined that the MMO values, which are known to vary on a population basis, were also normal MMO values in the population where this study was conducted [20]. In addition, it was observed that this MMO value was preserved at 6 months (T2). In these data, the functional effect of arthrocentesis and its contribution to pain were observed.

It is thought that occlusal splints provide a change in mechanical sensorial input originating from periodontal tissues and masticatory muscles, thereby reducing intra-articular pressure. For this reason, it is preferred in the nonsurgical treatment of TMJ diseases [12]. By the same mechanism, splint therapy is preferred in cases where stress and joint load increase, such as Bruxism. It has been shown in many studies to be beneficial in first-line treatment in patients with TMD disc problems. On the other hand, a report stated that splint treatment had no effect on patients with disc displacement [17]. In this study, it was observed that the MMO values of occlusal splint users and nonusers increased at T1 and T2, and the VAS scores decreased at T1 and T2. There was no difference between those who used and did not use a splint. In other words, splint therapy was not effective in terms of pain and MMO patency. Previous studies have shown that splint alone has no superiority in success compared to arthrocentesis [22,23]. On the other hand, one study stated that arthrocentesis and splint therapy were superior to splint therapy alone. Diracoğlu stated that arthrocentesis is superior to other traditional treatments in terms of pain control. In this study, pain relief at T1 was demonstrated by a decrease in the VAS score. Post-treatment anti-inflammatory drugs and muscle relaxants used on patients may have had an effect on this. However, in this study, it was observed that these drugs alone did not have any effect.

Muscle relaxants and anti-inflammatory drugs have an effect on pain control, muscle relaxation, and reduction in the release of inflammatory mediators in the acute phase. There are few publications about muscle relaxants used after arthrocentesis for this purpose [24,25]. In this study, since the drugs were used for a short time, the effect could only be measured at T1. Also, no significant difference was observed in terms of the MMO and VAS scores for those who used these drugs and those who did not.

Physical therapy is applied to provide negative loading and pain control in TMD patients. There are many studies on non-reduction disc displacement [26,27,28]. It was observed that physical therapy was effective in treatment, the MMO values increased, and the VAS score decreased [26]. In this study, no significant contribution to physical therapy was observed in terms of the MMO values and VAS scores of the patients. The reason for this can be attributed to the fact that the patients did not apply physical therapy alone effectively and that they stopped exercising due to the increase in joint movement after arthrocentesis.

Regardless of whether the locking was longer or shorter than 6 months, there was no significant difference in terms of the MMO and VAS scores in this study. Similar results have been observed before, but the 6-month results have not been reported before [27].

There are several limitations to this study. First of all, this study was retrospective, and it was conducted according to the results of the data. Patients with missing information in the records were not included in this study. A prospective and multi-participant study may provide more reliable data. The second problem was the follow-up time. Although this study was a rare study since it covered a long period of time of 6 months, studies with longer follow-ups may give more reliable findings about the efficacy of arthrocentesis.

In conclusion, it was observed that arthrocentesis therapy is an effective method in the treatment of temporomandibular joint diseases. It was shown that the increase in the MMO value was observed from the third day (T1) and that this increase was maintained in the sixth month (T2). It was observed that the pain decreased from T1, and this effect continued at T2. Splint, drug therapy, and physical therapy did not contribute to the benefit of arthrocentesis. It is obvious that more studies on this subject and an increase in samples will contribute to the literature.

## Figures and Tables

**Table 1:** Gender, age, and MMO distributions.

Gender (Participant (n))	Male (16)	Female (63)	*p* Value
Age (mean (SD))	35.4 (4.9)	36.7 (6.1)	0.57
MMO T0 (mm) (mean (SD))	35.7 (4.2)	35.4 (5.2)	0.71
MMO T1 (mm) (mean (SD))	42.1 (4.4)	41.3 (4.3)	0.67
MMO T2 (mm) (mean (SD))	42.0 (4.8)	41.6 (5.1)	0.35

*p* significance of the Wilcoxon matched pairs test. *p* < 0.05 was considered statistically significant. MMO maximum mouth opening. T0: Baseline (Before arthrocentesis). T1: 3 days after arthrocentesis. T2: 6 months after arthrocentesis.

**Table 2:** Results and comparison of the MMO and VAS score after arthrocentesis.

	T0	T1	T0–T1 (*p* Value)	T2	T0–T2 (*p* Value)
MMO (mean) (mm)	36.1	41.4	5.3 (0.01)	41.3	5.2 (0.002)
VAS (mean)	6.2	2.3	−3.9 (0.02)	2.1	−4.1 (0.03)

*p* significance of the Wilcoxon matched pairs test. *p* < 0.05 was considered statistically significant. MMO maximum mouth opening. VAS visual analog scale. T0: Baseline (Before arthrocentesis). T1: 3 days after arthrocentesis. T2: 6 months after arthrocentesis. T0–T1: Comparison of the T0 and T1. T0–T2: Comparison of the T0 and T2.

**Table 3:** Variation of the VAS score change according to the variables at baseline and on the 3rd day after arthrocentesis.

	VAS Score					
	Number	T0 (Mean (SD))	T1 (Mean (SD))	*p* Value *	Difference of VAS (Mean (SD))	*p* Value †
Appliance treatment						
No splint	34	4.41 (1.68)	1.87 (1.85)	<0.001	−2.54 (1.67)	0.238 a
Splint tx	45	5.11 (1.98)	1.76 (1.65)	<0.001	−3.35 (1.69)	
Dx						
Group 1	22	4.66 (1.65)	1.81 (1.54)	<0.001	−2.85 (1.76)	0.764 b
Group 2	45	4.55 (1.79)	1.76 (1.63)	<0.001	−2.79 (1.64)	
Group 3	12	4.52 (1.98)	1.72 (1.46)	<0.001	−2.8 (1.89)	
Muscle relaxant and anti-inflamatuar drug						
No	15	4.92 (2.65)	2.11 (1.76)	<0.001	−2.81 (1.55)	0.368 a
Yes	64	4.98 (2.76)	2.01 (1.65)	<0.001	−2.97 (1.75)	
Physical therapy						
No	18	4.61 (2.41)	2.31 (1.81)	<0.001	−2.30 (1.44)	0.266 a
Yes	61	4.88 (2.66)	2.01 (1.61)	<0.001	−2.87 (1.69)	
Locking time						
Less than 6 months	35	4.21 (1.67)	1.73 (1.45)	<0.001	−2.48 (1.98)	0.759 a
6 or more than 6 months	41	4.12 (1.87)	1.71 (1.56)	<0.001	−2.41 (1.87)	

*p* < 0.05 was considered statistically significant. DD—disc displacement, DJD—degenerative joint disease, * *p* value calculated via paired *t*-test. † Difference = post–pre. a *p* value calculated via *t*-test. b *p* value calculated via ANOVA. T0: Baseline (Before arthrocentesis). T0: Baseline (Before arthrocentesis). T1: 3 days after arthrocentesis. Group 1: DD with locking. Group 2: DD without locking. Group 3: Degenerative joint disease(DJD).

**Table 4:** Variation of VAS score change according to the variables at baseline and in the 6 months after arthrocentesis.

	VAS Score					
	Number	T1 (Mean (SD))	T2 (Mean (SD))	*p* Value *	Difference of VAS (Mean (SD))	*p* Value †
Appliance treatment						
No splint	34	4.41 (1.68)	1.90 (1.81)	<0.001	−2.54 (1.67)	0.231 a
Splint tx	45	5.11 (1.98)	1.87 (1.75)	<0.001	−3.35 (1.69)	
Dx						
Group 1	22	4.66 (1.65)	1.93 (1.74)	<0.001	−2.85 (1.76)	0.649 b
Group 2	45	4.55 (1.79)	1.86 (1.83)	<0.001	−2.79 (1.64)	
Group 3	12	4.52 (1.98)	1.82 (1.66)	<0.001	−2.8 (1.89)	
Muscle relaxant and anti-inflamatuar drug						
No	15	4.92 (2.65)	2.31 (1.71)	<0.001	−2.81 (1.55)	0.887 a
Yes	64	4.98 (2.76)	2.11 (1.61)	<0.001	−2.97 (1.75)	
Physical therapy						
No	18	4.61 (2.41)	2.31 (1.81)	<0.001	−2.30 (1.44)	0.561 a
Yes	61	4.88 (2.66)	2.01 (1.61)	<0.001	−2.87 (1.69)	
Locking time						
Less than 6 months	35	4.21 (1.67)	1.79 (1.25)	<0.001	−2.48 (1.98)	0.894 a
6 or more than 6 months	44	4.12 (1.87)	1.78 (1.46)	<0.001	−2.41 (1.87)	

*p* < 0.05 was considered statistically significant. DD—disc displacement, DJD—degenerative joint disease, * *p* value calculated via paired *t*-test. † Difference = post–pre. a *p* value calculated via *t*-test. b *p* value calculated via ANOVA. T0: Baseline (Before arthrocentesis). T1: 3 days after arthrocentesis. T2: 6 months after arthrocentesis. Group 1: DD with locking. Group 2: DD without locking. Group 3: Degenerative joint disease(DJD).

**Table 5:** Variation of MMO value change according to the variables at baseline and on the 3rd day after arthrocentesis.

	MMO					
	Number	T0 (Mean (SD))	T1 (Mean (SD))	*p* Value *	Difference of MMO (Mean (SD))	*p* Value †
Appliance treatment						
No splint	34	36.1 (5.8)	41.8 (6.8)	<0.001	−5.7 (3.7)	0.339 a
Splint tx	45	36.9 (7.2)	42.1 (5.9)	<0.001	−5.2 (4.9)	
Dx						
Group 1	22	34.2 (5.5)	42.5 (6.4)	<0.001	−8.3 (4.6)	0.603 b
Group 2	45	37.1 (4.7)	42.9 (5.1)	<0.001	−5.8 (4.7)	
Group 3	12	37.2 (5.2)	41.7 (6.1)	<0.001	−4.5 (4.7)	
Muscle relaxant and anti-inflamatuar drug						
No	15	36.3 (5.1)	41.9 (4.5)	<0.001	−5.6 (4.3)	0.692 a
Yes	64	37.8 (5.1)	42.1 (4.9)	<0.001	−4.3 (3.7)	
Physical therapy						
No	18	34.7 (5.5)	42.8 (5.1)	<0.001	−8.1 (4.6)	0.467 a
Yes	61	37.0 (4.6)	42.1 (5.2)	<0.001	−5.1 (4.1)	
Locking time						
Less than 6 months	35	36.5 (6.7)	41.3 (5.2)	<0.001	−4.8 (1.9)	0.549 a
6 or more than 6 months	41	36.7 (5.8)	42.7 (6.1)	<0.001	−6 (3.7)	

*p* < 0.05 was considered statistically significant. DD—disc displacement, DJD—degenerative joint disease, * *p* value calculated via paired *t*-test. † Difference = post–pre. a *p* value calculated via *t*-test. b *p* value calculated via ANOVA. T0: Baseline (Before arthrocentesis). T0: Baseline (Before arthrocentesis). T1: 3 days after arthrocentesis. Group 1: DD with locking. Group 2: DD without locking. Group 3: Degenerative joint disease(DJD).

**Table 6:** Variation of MMO value change according to the variables at baseline and in the 6 months after arthrocentesis.

	MMO					
	Number	T1 (Mean (SD))	T2 (Mean (SD))	*p* Value *	Difference of MMO (Mean (SD))	*p* Value †
Appliance treatment						
No splint	34	36.1 (5.8)	43.2 (6.3)	<0.001	−7.1 (4.7)	0.492 a
Splint tx	45	36.9 (7.2)	42.6 (6.2)	<0.001	−5.7 (5.1)	
Dx						
Group 1	22	34.2 (5.5)	42.7 (6.2)	<0.001	−8.5 (5.5)	0.598 b
Group 2	45	37.1 (4.7)	43.1 (4.1)	<0.001	−6 (4.9)	
Group 3	12	37.2 (5.2)	42.9 (5.1)	<0.001	−5.7 (5.2)	
Muscle relaxant and anti-inflamatuar drug						
No	15	36.3 (5.1)	42.6 (5.1)	<0.001	−6.3 (4.4)	0.398 a
Yes	64	37.8 (5.1)	42.9 (5.9)	<0.001	−5.1 (4.1)	
Physical therapy						
No	18	34.7 (5.5)	42.3 (6.1)	<0.001	−7.6 (4.6)	0.544 a
Yes	61	37.0 (4.6)	42.9 (5.2)	<0.001	−5.9 (4.3)	
Locking time						
Less than 6 months	35	36.5 (6.7)	42.7 (5.2)	<0.001	−6.2 (3.9)	0.889 a
6 or more than 6 months	41	36.7 (5.8)	43.1 (6.1)	<0.001	−6.4 (4.3)	

*p* < 0.05 was considered statistically significant. DD—disc displacement, DJD—degenerative joint disease, * *p* value calculated via paired *t*-test. † Difference = post–pre. a *p* value calculated via *t*-test. b *p* value calculated via ANOVA. T0: Baseline (Before arthrocentesis). T1: 3 days after arthrocentesis. T2: 6 months after arthrocentesis. Group 1: DD with locking. Group 2: DD without locking. Group 3: Degenerative joint disease(DJD).

## Data Availability

Not applicable.
